# Machine Learning in Prostate MRI for Prostate Cancer: Current Status and Future Opportunities

**DOI:** 10.3390/diagnostics12020289

**Published:** 2022-01-24

**Authors:** Huanye Li, Chau Hung Lee, David Chia, Zhiping Lin, Weimin Huang, Cher Heng Tan

**Affiliations:** 1 School of Electrical and Electronic Engineering, Nanyang Technological University, Singapore 639798, Singapore; li0002ye@e.ntu.edu.sg (H.L.); ezplin@ntu.edu.sg (Z.L.); 2 Department of Diagnostic Radiology, Tan Tock Seng Hospital, Singapore 308433, Singapore; chau_hung_lee@ttsh.com.sg; 3 Department of Radiation Oncology, National University Cancer Institute (NUH), Singapore 119074, Singapore; david_chia@nuhs.edu.sg; 4 Institute for Infocomm Research, A*Star, Singapore 138632, Singapore; wmhuang@i2r.a-star.edu.sg; 5 Lee Kong Chian School of Medicine, Nanyang Technological University, Singapore 639798, Singapore

**Keywords:** prostate MRI, cancer, deep learning, machine learning, PI-RADS, segmentation, detection, registration, diagnosis, survey

## Abstract

Advances in our understanding of the role of magnetic resonance imaging (MRI) for the detection of prostate cancer have enabled its integration into clinical routines in the past two decades. The Prostate Imaging Reporting and Data System (PI-RADS) is an established imaging-based scoring system that scores the probability of clinically significant prostate cancer on MRI to guide management. Image fusion technology allows one to combine the superior soft tissue contrast resolution of MRI, with real-time anatomical depiction using ultrasound or computed tomography. This allows the accurate mapping of prostate cancer for targeted biopsy and treatment. Machine learning provides vast opportunities for automated organ and lesion depiction that could increase the reproducibility of PI-RADS categorisation, and improve co-registration across imaging modalities to enhance diagnostic and treatment methods that can then be individualised based on clinical risk of malignancy. In this article, we provide a comprehensive and contemporary review of advancements, and share insights into new opportunities in this field.

## 1. Introduction

Prostate MRI has been developed into an important tool for the management of prostate cancer (PCa). It is recommended as the first-line screening method for patients with a clinical suspicion of prostate cancer [1]. The Prostate Imaging-Reporting and Data System (PI-RADS) represents a comprehensive set of guidelines, standardized observations and lexicon, which aims to stratify probability of clinically-significant prostate cancer (csPCa) on MRI [2].

Prostate MRI and PI-RADS aim to guide biopsy by identifying the most suspicious areas within the prostate to be targeted. MRI-guided targeted biopsy pathways have been shown to improve the detection of clinically significant prostate cancer with a reduction in the number of biopsy cores, compared to conventional systematic biopsy [3,4]. Improved prostate cancer localization with MRI has facilitated investigations into focal therapy, such as via cryotherapy, high-intensity focused ultrasound (HIFU) and brachytherapy. Focal therapy is a promising option that is being investigated as a possible alternative to whole-gland treatment (prostatectomy or radiotherapy) in patients with low-volume disease, and avoids adverse effects such as incontinence, erectile dysfunction and radiation enteritis [5].

Despite vast improvements in MRI techniques, there are still limitations to prostate MRI interpretation in clinical practice, and experience is still accumulating. This is evident from the fact that PI-RADS has undergone two revisions since version 1 was introduced in 2012 (version 2 in 2015 and version 2.1 in 2019). However, differentiating cancer from non-cancerous pathology such as benign prostatic hyperplasia or inflammation on MRI remains challenging [6,7]. Considerable inter-observer variability also remains, even in the latest PI-RADS version 2.1 [8,9]. Furthermore, variations in scanning parameters and image quality between scanners make it difficult to accurately compare between MRI studies [10].

There have been efforts to address these limitations. Techniques such as quantitative MRI analysis and computer-aided diagnosis enable a more objective analysis of prostate MRI and have been shown to improve diagnostic accuracy and reproducibility [11,12,13]. In recent years, there has been increasing interest in the use of artificial intelligence (AI) and machine learning (ML) in radiology.

Recent studies suggest that applying ML in prostate MRI could improve diagnostic accuracy and reduce inter-reader variability by highlighting suspicious areas on MRI, allowing a more focused interpretation by the radiologist during conventional scan interpretation [14]. ML has also been shown to be able to predict lesion aggressiveness and treatment response [15,16]. Several studies have demonstrated comparable performances between ML and expert radiologists in head-to-head comparisons for MRI interpretation [17,18].

The value of ML in prostate MRI could go beyond imaging interpretation and diagnosis. For example, in MRI–US fusion targeted prostate biopsy, the precise gland and region-of-interest segmentation and image co-registration between MRI and US are important for optimal biopsy. Segmentation and image fusion are usually manually performed, which can be laborious, time-consuming, and subject to inter-operator variation. ML has shown potential in gland segmentation and MRI–US fusion in terms of accuracy and efficiency [19,20]. This can be further extrapolated to radiation therapy planning and possible focal therapy, where precise segmentation is necessary to optimize dose to region of interest and reduce injury to adjacent normal prostate tissue [21].

Prostate MRI interpretation is generally recognized to present a steep learning curve [22]. Through automation, ML potentially enables more consistent interpretation across readers with various experience levels, improving inter-reader agreement, and reducing the need for expert training in prostate MRI interpretation. This would particularly benefit surgeons and radiotherapists who normally do not receive formal radiology training, when it comes to the management of prostate cancer patients.

## 2. Machine Learning Applications to Enhance Utility of Prostate MRI: Current Status

### 2.1. Related Reviews

A review in 2019 [16] has demonstrated the capability of machine learning (ML) and deep learning (DL) to process prostate MRI during different tasks, including segmentation, cancer detection, cancer assessment, local staging, and biochemical recurrence. However, the summary is limited and covered fewer methods published up to 2019. Others, for example, Chaddad et al. [23], discussed the existing clinical applications, as well as machine learning-based studies weighted more on the prostate MRI radiomics pipeline and methods of cancer grade prediction on the Gleason score. Another review by Zeeshan et al. [24] introduced the literature using AI to support urological disease treatments, with only a small amount of research based on prostate MRI.

In this section, we will review the literature, studying the ML and DL applications in prostate MRI segmentation, registration, lesion detection and scoring, and treatment decision support, over a wider range in time. More importantly, both traditional and recently published studies will be covered, especially focusing on clinic tasks, methods utilized, data, and results. 

### 2.2. Segmentation

**Figure 1:**
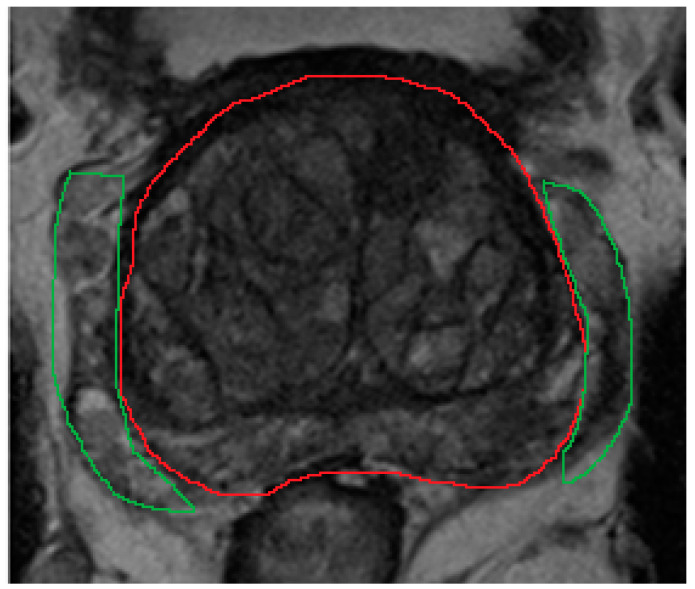
On MRI, the periprostatic venous plexus appears as serpinginous hyperintense structures with foci of signal voids adjacent to the prostate (green outline), and can be closely related to the prostate capsule (red outline). It may have similar heterogeneous appearance as the peripheral zone. Therefore, during manual segmentation, it can be mistaken as part of the prostate to less experienced operators.

The aim of segmentation is to define the boundary of the prostate gland, prostate zones (central, transition, peripheral zones), and any focal lesions. Gland and lesion segmentation is important when performing fusion-based targeted biopsy or focal therapy, as these clinical settings require the accurate delineation of prostate, zonal, and lesion contours. Segmentation can be performed either manually or by ML/DL methods. In practice, manual segmentation can be time-consuming and subjective, depending on the experts’ perception and level of experience. This can range from highly accurate when delineating the transition zone from the peripheral zone, to highly subjective and variable when delineating the prostate margins from periprostatic venous plexus in the mid-gland to apex regions (Figure 1). 

Therefore, interest in developing accurate automatic segmentation tools has rapidly increased. A comprehensive review [25] summarized recent ML and DL applications for prostate MRI segmentation until December 2020, showing the important and mature status for ML in automatic prostate MRI segmentation tasks. In this paper, we will revisit the main methods by which segmentation can be performed by ML and DL, and review more recent research work covered. The papers mentioned in this section are summarised in Table 1. 

The evaluating metric for segmentation is usually expressed as Dice similarity coefficient (DSC), measuring the degree of overlap between the predicted and the true masks [26]. 

**Table 1:** Machine learning-based segmentation methods for prostate MRI. The abbreviations are shown below ^1^.

Publication Year	Method	Prostate Zone	Input Image Dimension (Pixel/Voxel/mm)	Data Source	MRI Sequence(s)	Sample Sizes			CV	Results		Refs.
						Train	Val	Test		Acc (%)	DSC (%)	
2008	Nonrigid registration of prelabelled atlas images	WG	512 × 512 × 90, 271 × 333 × 86	Pv	T2w	38	-	50	No	-	85	[27]
2009	Level set	WG		Pv	DWI	10	-	10	No	-	91	[28]
2012	AAM	WG	0.54 × 0.54 × 3 mm	Pv	T2w	86	-	22	5-fold		88	[29]
2007	Organ model-based, region-growing	WG	3D	Pv	T1w, T2w	15	-	24	No	94.75		[30]
2014	RF and graph cuts	WG	512 × 512 or 320 × 320	PRO12	T2w	50	-	30	10-fold	-	>91 (training), >81 (test)	[31]
2014	Atlas-based AAM and SVM	WG	512 × 512	Pv	T2w	100	-	40	leave-one-out	90	87	[32]
2016	Atlas and C-Means classifier	WG, PZ, TZ	Varying sizes	PRO12, Pv	T2w	30	35		No	-	81 (WG), 70 (TZ), 62 (PZ)	[33]
2016	Volumetric CNN	WG	128 × 128 × 64	PRO12	T2w	50	-	31	No	-	86.9	[34]
2017	FCN	WG, TZ	0.625 × 0.625 × 1.5 mm	PRO12	T2w	50	-	30	10-fold	-	89.43	[35]
2021	V-Net using bicubic interpolation	WG	1024 × 1024 × 3 × 16	PRO12, Pv	T2w	106	-	30	Y	-	96.13	[36]
2019	Cascade dense-UNet	WG	256 × 256	PRO12	T2w	40	-	10	5-fold	-	85.6	[37]
2021	3D-2D UNet	WG	-	Pv	T2w	299	-		5-fold	-	89.8	[38]
2020	convLSTM and GGNN	WG	28 × 28 × 128	PRO12, ISBI13, Pv	T2w	140	-	30	No	-	91.78	[39]
2020	Transfer learning, data augmentation, fine-tuning	WG, TZ	-	Pv	T2w	684	-	406	10-fold	-	91.5 (WG), 89.7 (TZ)	[40]
2021	Federated learning with AutoML	WG	160 × 160 × 32	MSD-Pro, PRO12, ISBI13, PROx	T2w	344	46	96	No	-	89.06	[41]
2020	Anisotropic 3D multi-stream CNN	WG	144 × 144 × 144	PRO12, Pv	T2w	87	30	19	4-fold	-	90.6 (base), 90.1 (apex)	[42]
2020	MS-Net	WG	384 × 384	Pv	T2w	63	-	16	No	-	91.66	[43]
2017	FCN	WG, TZ	144 × 144 × 26	PRO12, Pv	DWI	141	-	13	4-fold	97	93,88	[44]
2020	Transfer learning	WG, TZ	1.46 × 1.46 × 3 mm	Pv	DWI	291	97	145	No	-	65 (WG), 51 (TZ)	[45]
2019	Cascaded U-Net	WG, PZ	192 × 192	Pv	DWI	76	36	51	No	-	92.7 (WG), 79.3 (PZ)	[46]
2021	Three 3D/2D UNet pipeline	WG, PZ, TZ	256 × 256 × (3 mm)	Pv	T2w	145	48	48	No	-	0.94 (WG), 0.914 (TZ), 0.776 (PZ)	[47]
2021	U-Net, ENet, ERFNet	WG, PZ, TZ	512 × 512	PROx	T2w	99	-	105	5-fold	-	ENet (best): 91 (WG), 87 (TZ), 71 (PZ)	[48]
2021	Transfer learning, aggregated learning, U-Net	WG, PZ, CG	192 × 192, 192 × 192 × 192	ISBI13	T2w	5–40	-	20	5-fold	-	73 (PZ), 83 (CG), 88 (WG)	[49]
2018	PSNet	WG	320 × 320 × 512 × 512	PRO12, ISBI13	T2w	112	-	28	5-fold	-	85	[50]

^1^ Val = validation, CV = cross–validation, Acc = accuracy, DSC = dice similarity coefficient, Refs. = reference, - = not reported. For datasets, Pv = private, PRO12 = PROMISE12 [51], ISBI13 = NCI-ISBI 2013 Challenge [52], MSD-Pro = MSD Prostate [53], PROx = PROSATETx Challenge [15]. For prostate zones, WG = whole gland, TZ = transition zone, PZ = peripheral zone.

#### 2.2.1. Traditional Machine Learning Methods

Traditional segmentation methods can be summarized as atlas-based models, deformable models, and feature-based ML methods. “Atlas” is a collection of manually segmented structures, which serves as a reference to be registered with additional or new patient images on longitudinal follow-up. A study in 2008 [27] applied the inter-subject registration of Atlas images for pelvic MR images. The deformed images were fused using various voting techniques, to generate a consensus label for the segmentation of new patients’ images, resulting in a DSC of 0.85. Deformable models, like level sets [28] and active appearance models (AAMs) [29], make use of prior geometric or statistical knowledge for segmentation. In 2007, Pasquier et al. [30] applied a statistic model for prostate gland segmentation and region-growing methods for rectum and bladder segmentation. In 2009, Liu et al. [28] used prior shape information for level set, a numerical technique, yielding a mean DSC of 0.91. Another approach uses feature-based machine learning methods to cluster the image into prostate and background regions. ML classifiers, such as random forest (RF) and support vector machine (SVM), widely adopted for binary or multi-class classification are applied here for assigning membership values to either the target or background group. For example, Mahapatra et al. [31] used two sets of RF for the identification of super-voxels and the classification of prostate voxel using surrounding features. A graph cut method is used based on the RF output as cost function, resulting in a Dice metric of more than 0.81 on the test set. 

Hybrid methods are also common, which combine different models, including some of the aforementioned methods, to achieve improved performance. For instance, in 2014, Cheng et al. [32] combined atlas-based AAM for registration, and SVM for classification, to achieve a relatively high segmentation result (accuracy 90%) on prostate delineation. Later, in 2016, Chilali et al. [33] developed a prostate and zonal segmentation model using atlas images and C-means clustering, achieved mean Dice accuracy values of 0.81, 0.70, and 0.62, for the prostate, the transition zone, and peripheral zone segmentation, respectively. Regardless of their differences, all these methods ultimately required manual ground truth segmentation by experts, in order to achieve reasonably accurate modelling results.

#### 2.2.2. Deep Learning-Based Methods

Recently, deep convolution neural networks (deep CNNs) for prostate segmentation have been developed, and demonstrate better segmentation accuracy than using conventional ML methods [25,54]. This is due to their ability to learn the appropriate features of input images, and their enhancement with data augmentation methods that provide more data to train the network. The review [25] classified the DL algorithms into four groups according to the segmentation techniques used. They are: feature encoder, up-sampling, the resolution increment of features, and regional proposal-based techniques. Another review published in 2020 [55] listed the deep learning-based prostate MRI and CT segmentation methods, with fewer papers covered than in [25] (19 vs. 110) for MRI segmentation. The papers were classified based on methods applied through the DL-based segmentation process (e.g., data pre-processing, loss function, optimizer, ground truth, post processing). 

The fully convolutional network (FCN) [56] is the most widely used method for semantic segmentation with the employment of skip connection, pooling, and up-sampling. U-Net [57] is a further extension of FCN. With a regular CNN followed by up-sampling that increases the size of the feature map, it demonstrated striking performance, and has been widely used in prostate and zonal segmentation. In 2016, Milletari et al. [34] took the idea of U-Net architecture to develop a volumetric FCN (V-Net), to segment prostate volumes from MRI. To date, many novel variations of deep learning have been applied to improve the segmentation performance by modifying FCN, U-Net, or V-Net. For example, Yu et al. [35] introduced the long and short residual connections into 3D FCN, Tian et al. [50] fine-tuned an FCN that has been pre-trained using a large dataset, and Li et al. [37] added dense blocks and transition layers to two cascaded U-Net models. Recently, Jin et al. [36] improved V-Net using bicubic interpolation and achieved DSCs of 0.975 and 0.983 on two public datasets. Ushinsky et al. [38] introduced a novel 3D-2D U-Net CNN which was trained on 3D images, and completed object detection and segmentation on 2D images, resulting in a DSC of 0.898. 

When performing segmentation, the multi-modal 3D MR images can either be used directly or cropped into 2D slices as input for the DL model. Neither approach is without limitations. Splicing the 2D segmentation from 3D results may cause jaggedness and faults, whereas the amount of 3D MRI data is usually not sufficient to train the deep 3D neural network, leading to struggles in increasing segmentation accuracy. 

To learn about better correlation between neighbour image slices in 2D segmentation, Tian et al. [39] proposed a convolutional long short-term memory model (convLSTM), which enables the model to capture the spatial relation between neighbouring vertices. To solve the problem of a lack of MRI data for a deep learning network, several methods have been proposed. Sanford et al. [40] showed that the combination of transfer learning, data augmentation, and test time fine-tuning could benefit prostate segmentation. Roth et al. [41] combined federated learning (FL) and automated machine learning (AutoML) [58] to increase data diversity, achieving an average Dice score of 0.89. Meyer et al. [42] proposed an anisotropic multi-stream segmentation CNN to process additional scan directions (dual-plane and triple-plane), rather than simply axial scans, which significantly improved (*p* < 0.05) over segmentation on a plain axial view (base DSC 0.906 vs. 0.898, apex DSC 0.901 vs. 0.888). Liu et al. [43] proposed an MS-Net for segmentation from multiple sources of data using a knowledge transfer method. An overall AUC of 0.9166 was achieved across three sites. 

Amongst the myriad of possible approaches, we anticipate that FCN-based U-Net architecture will continue to be applied as a backbone in most segmentation tasks, while data augmentation and transfer learning could effectively improve segmentation performance. That said, head-to-head comparisons of some of the more robust and mature methods will become necessary in the future. 

#### 2.2.3. Zonal Segmentation

While the majority of current works have focused on whole prostate gland segmentation, some other studies have focused instead on the internal structure of the prostate, such as the delineation between the peripheral zone (PZ) and the transition zone (TZ). PZ is the origin of most carcinomas, while cancers in TZ, though less common, are more difficult to detect, due to concomitant benign prostatic hyperplasia (BPH). This has led to distinct clinical evaluation criteria for the different zones on the widely used PI-RADS. In order to train machines towards accurately segmenting tumours, it is necessary to segment different zones inside the prostate gland first. 

Clark et al. [44] combined VGG ConvNet [59] and U-Net-based architecture for whole gland (WG) and TZ segmentation. Motamed et al. [45] applied transfer learning and fine-tuning on a modified U-Net architecture for WG and TZ segmentation. Zhu et al. [46] used K-means clustering for coarse segmentation, and a cascaded U-Net model for WG and PZ segmentation. The algorithm achieved higher DSC than U-Net (0.93 vs. 0.87 and 0.79 vs. 0.67 for WG and PZ, respectively). In 2021, Bardis et al. [47] used a three 3D-2D U-Net models pipeline to first localize the prostate square shape zone, then segment prostate WG and PZ vs. TZ, achieving DSC 0.94, 0.914 and 0.776 for WG, TZ and PZ, respectively. 

**Figure 2:**
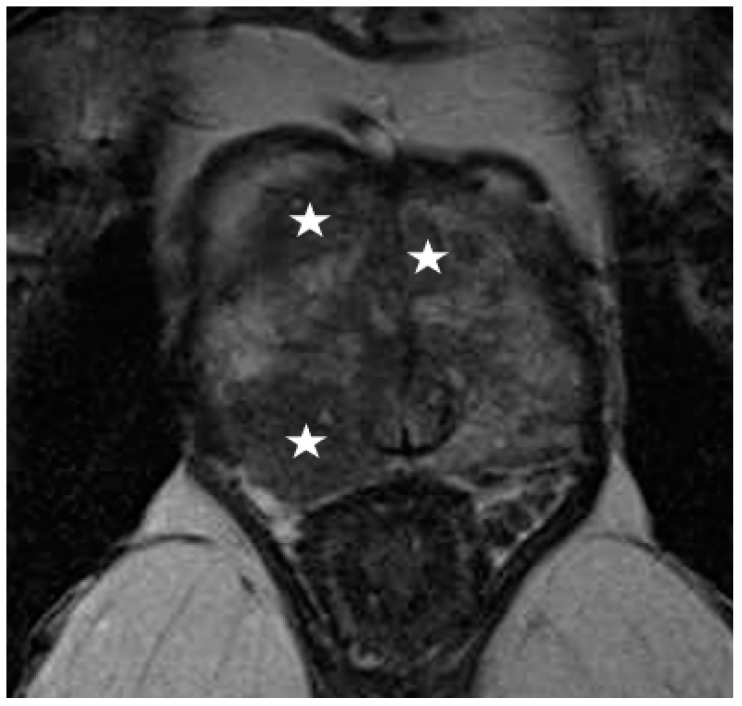
Multifocal prostate cancer seen as hypointense lesions on T2-weighted imaging (star), obscuring the boundaries between peripheral and transition zone, making zonal segmentation challenging.

**Figure 3:**
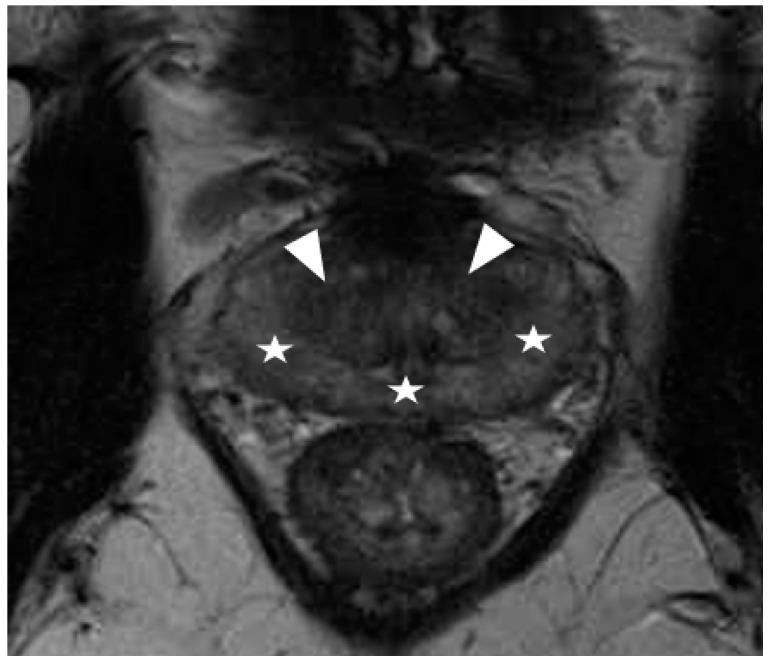
Prostatitis typically appears as diffuse hypointensity in the peripheral zone on T2-weighted imaging (star), resulting in an almost similar signal to stromal nodules related to benign prostatic hyperplasia in the transition zone (arrowhead). This may make differentiation between peripheral and transition zone difficult, and zonal segmentation challenging.

**Figure 4:**
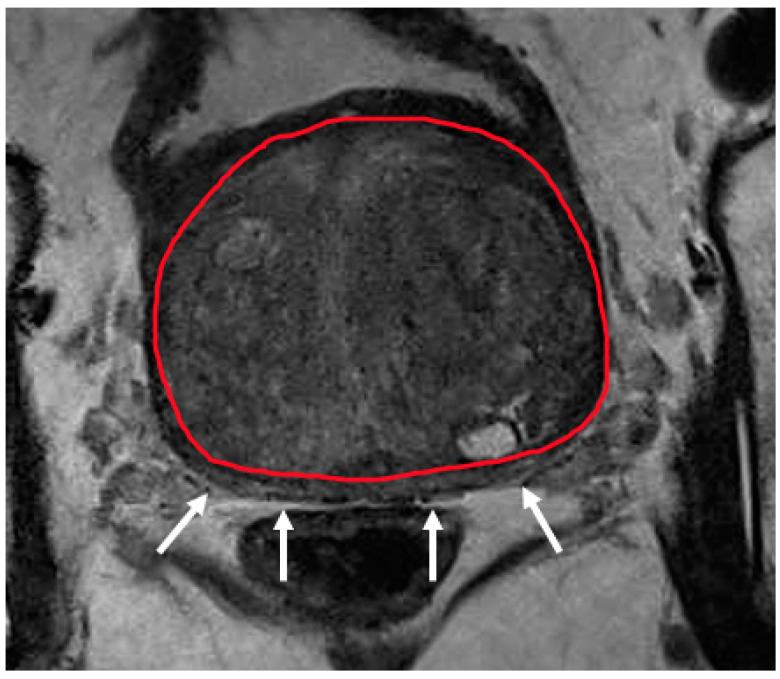
Severe hypertrophy of the transition zone (segmented in red) compressing on the peripheral zone which appears as a thin sliver (white arrows). Reduced visualisation of the peripheral zone in this case can make zonal segmentation challenging.

Recently, several studies have compared different deep CNN models on zonal segmentation. Cuocolo et al. [48] compared three deep learning methods, UNet, an efficient neural network (ENet) [60], and efficiently residually factorize ConvNet (ERFNet) [61] in the PROSTATEx public dataset. ENet (0.91, 0.87, 0.71) and UNet (0.88, 0.86, 0.70) were more accurate than ERFNet (0.87, 0.84, 0.65) in terms of DSC (for WG, TZ and PZ, respectively), while ENet outstood the other two methods, with faster convergence speed and fewer parameters. Saunders et al. [49] compared the performance of independent training, transfer learning, and aggregated learning based on 3D and 2D U-Net models, on the premise of limited training data. In addition, 3D U-Net was found to be more robust to a small sample size (five training cases) than 2D U-Net by an average DSC of 0.18, while transfer learning and aggregated learning (similar DSC: 0.73, 0.83, 0.88 for PZ, CG, WG, respectively) both outperformed independent training (DSC 0.65, 0.77, 0.83) when using five internal training cases. Predictably, automated segmentation between PZ and TZ can become challenging in cases where tumours span across both zones, since false positives like prostatitis in the PZ reduce its normal high T2 signal to become isointense to the TZ, whereas severe benign prostatic hypertrophy in the TZ compresses the PZ, reducing the ability to discriminate between the two zones (Figure 2, Figure 3 and Figure 4). 

### 2.3. Image Registration

The aim of image registration is to transform different types of images into the same coordinates to ensure spatial correspondence. For interventions such as transrectal biopsy, focal therapy and high dose rate (HDR) brachytherapy procedures, the fusion of MR images with real-time ultrasound images facilitates accurate localisation for needle placement [62]. For patients who have undergone radical prostatectomy, correlating pre-surgical MRI with whole-mount histopathology images could provide high-resolution information on the extension of cancer [63]. Moreover, dominant intraprostatic lesion (DIL) delineation from MRI (mpMRI) to CT images during radiation therapy also needs accurate MRI–CT image registration. 

For feature-based registration, the relatively sparse features need to be consistently matched between two imaging modalities. Manually obtaining landmarks from both modalities is strenuous and often impossible during intervention. Cognitive fusion is possible, but it generates a steep learning curve for procedurists. Meanwhile, further research is required to determine if software-based fusion is superior to cognitive fusion [64]. 

To date, ML- and DL-based approaches have been applied to the task of registration between mpMRI and other types of images. We will discuss the three most popular areas, MRI–ultrasound (MRI–US), MRI–histopathology, and MRI–CT registration. The studies mentioned in this section are summarised in Table 2. For quantitative validation, target registration error (TRE) is the most frequently used measurement, calculated as the root-mean-square distance over all pairs of corresponding landmarks in the registration image pairs for each patient. There are three types of image registration (rigid, affine, and deformable registration) depending on the transformations applied [65].

#### 2.3.1. MRI–US

**Figure 5:**
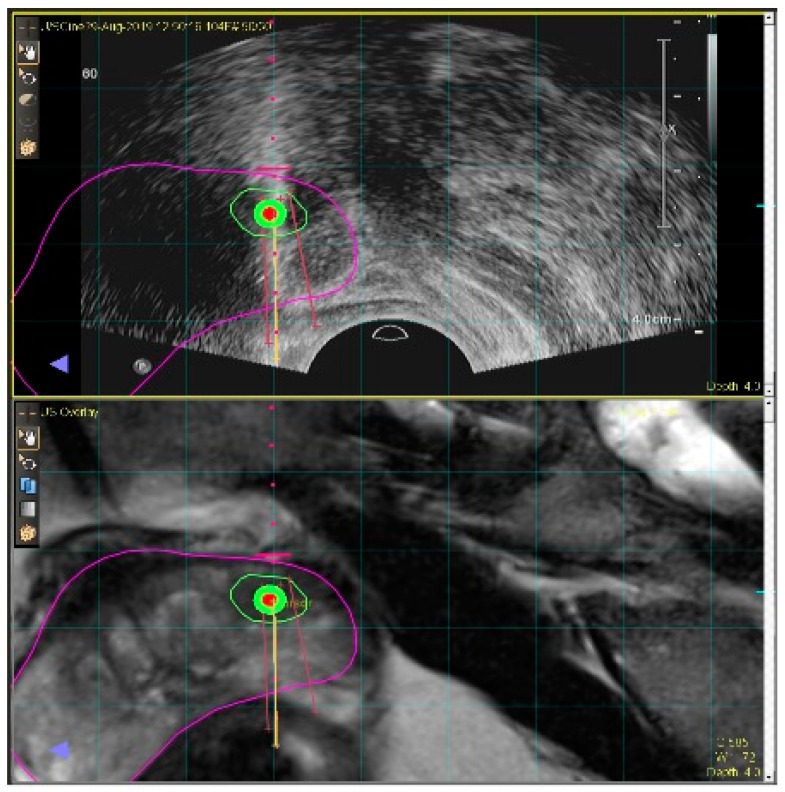
MRI–US fusion technique for targeted prostate biopsy requires precise registration between pre-operative prostate MRI (bottom image) and real-time ultrasound (top image).

Generally, transrectal (TR), or transperineal (TP), ultrasound images serve as the main guidance for prostate biopsies. However, they display poor contrast between healthy and cancerous regions. On the other hand, MRI, or mpMRI, is currently the mainstay for the detection and localization of PCas, and MRI-guided targeted biopsy pathways have been shown to increase the detection of clinically significant prostate cancer [4]. However, in-bore MRI-based interventions are cumbersome and consume valuable magnet scan time. As a result, MRI–US fusion techniques are increasingly favoured (Figure 5).

Traditional ML approaches for MR-US registration have mainly been based on biomechanical models and statistical deformation models. In 2002, Mohamed et al. [66] first proposed a finite-element (FE) analysis to simulate the prostate deformation model induced by the transrectal ultrasound probe. Principal component analysis (PCA) and linear least square fitting were applied to construct deformed prostate shapes from CT or MRI. Thereafter, Hu et al. [67,68] incorporated biomechanical parameters with random sampling and PCA. However, the randomly sampled biomechanical parameters may greatly differ from the real values of the patient, and hence, may not be appropriate for general usage. Subsequently, Wang et al. [69] proposed a patient-specific deformable registration model using a hybrid surface point matching method. Their model achieved a TRE around 1.44 mm. Nevertheless, all the above-mentioned methods relied on rigid surface registration and required prostate surface segmentation for initialization, while the biomechanical models are not readily available for practical clinical application.

Recently, deep learning models have been introduced for image registration. The principle is using the fixed and moving images to train the deep learning network to predict the appropriate transformation matrix between them. However, supervised deep learning is currently difficult to apply here, due to the unavailability of ground truth deformation. 

To tackle this issue, ongoing research focuses on weakly supervised or unsupervised deep learning models. Hu et al. [70,71] proposed a weakly supervised learning method with CNN using manually labelled anatomical structures. Yan et al. [72] proposed a multi-modality generative adversarial network (GAN) network. An unsupervised DL network enabled the simultaneous training of CNNs for transformation parameter estimation and registration quality evaluation. Zeng et al. [73] proposed a weakly supervised MRI–transrectal ultrasound (MRI–TRUS) registration method that first segmented MRI and TRUS prostate, and then applied affine and non-rigid registration using FCN, 2D CNN and 3D U-Net. The method produced a mean TRE of 2.53 mm. Similarly, Chen et al. [74] in 2021 also employed a segmentation model before the registration of MR images to the treatment planning US images after the needle insertion for HDR brachytherapy. 

To overcome the problem of rigid alignment error and organ deformation in real-time intervention, Bhardwaj et al. [75] proposed a 2D/3D DL-based constraint registration for real-time rigid and deformable error correction, achieving a TRE of 2.988 mm on clinical data. Biomechanical properties combined with DL networks also demonstrated effectiveness in several studies. Hu et al. [76] applied biomechanical FE simulations to regularize the registration in an adversarial learning approach, resulting in a registration error of 6.3 mm and a DSC of 0.82. Yang et al. [77] proposed a framework that first segmented the MRI and TRUS images, and then applied a point-cloud-based network with a built-in biomechanical constraint for registration. The model achieved a 1.57 mm TRE and a 0.94 DSC, approaching what could be implemented into a clinical routine. It is predicted that these techniques will significantly advance our ability to perform the accurate placement of needles for tissue sampling or local ablation (such as cryotherapy), where MR images can be a used as ground-truth, and fused with real-time TRUS, on which considerable deformation of the gland occurs during the procedure.

#### 2.3.2. MRI–Histopathology 

For patients who have undergone radical prostatectomy (RP), the whole-mount histopathology images can be correlated with pre-surgical MRI, such that cancers (and their attending Gleason grades) are accurately mapped. Developing such mapping between histopathology images and MRI could improve the existing MRI interpretation, as well as facilitating the machine learning methods to identify prostate cancer on MRI by providing accurate cancer labels, which will be introduced in the next section. Rusu et al. [79] developed RAPSODI for the registration of radiology and pathology images. The framework created a digital representation of tissue from the histopathology specimen to provide cancer labels for MRI. In 2020, Shao et al. [81] applied mono-modal MR and histopathology image pairs to a DL network for the estimation of affine and deformable transformation, which was then applied for mapping the cancer labels onto MRI. The method performed similarly to RAPSODI. However, these strategies assumed slice-to-slice correspondence between histopathology and MR images, which would require a significant alteration to the clinical workflow, which is typically not practiced in most centres performing RP. Sood et al. [82] introduced a novel framework without the need for slice-to-slice correspondence using a GAN-based network. The learned information from 3D MRI and histopathology slices was applied for mapping the extent of cancer onto MRI. The model achieved a 3D DSC of 0.95 for prostate gland and 0.68 for cancer. Advancements in this domain will invariably allow for a better understanding of treatment response to local ablative therapies, where the intent is to concentrate treatment dose to the tumour site, and spare the non-tumorous areas. 

#### 2.3.3. MRI–CT

Co-registration MRI with computed tomography (CT) is promising for radiation therapy planning and delivery for patients with prostate cancer. This method would combine the superior soft tissue delineation by MRI with the lower cost CT-based linear accelerator (Linac) units for optimal dose delivery in a localized disease. In 2019, Ghazal et al. [78] used RF based on an auto-context model to create synthetic CT images from MR images for dose calculations, resulting in more than 99% pass rate in gamma analysis. Recently, Fu et al. [80] used a 3D point cloud matching network after segmentation of MR and CBCT for registration. Their TRE was 2.68 mm, and the DSC was 0.93. In practice, there is a relatively urgent need for the clinical implementation of this technique, given the substantially higher cost of MRI-based Linacs, and the increasingly advanced capability of current radiotherapy machines in delivering modulated treatment doses to reduce the detrimental side effects of regional non-tumorous tissue damage. ML-/DL-based techniques in these clinical settings would also be particularly useful to facilitate treatment planning and diagnosis for surgeons, pathologists and radiotherapists who do not usually receive formal radiology training, and may not be familiar with MRI interpretation.

### 2.4. Lesion Detection and Characterization 

Ultimately, the optimal clinical treatment of prostate cancer requires the accurate grading and staging of disease. Prostate cancers can be divided into clinically significant and clinically insignificant cancers based on grade (Gleason score) and aggressiveness [83]. Clinically significant cancers (csPCa) would usually require definitive treatment, such as surgery or radiotherapy, while clinically insignificant cancers can be managed with active surveillance. Currently, the prostate imaging-reporting and data system (PI-RADS) is the main scoring system to indicate the probability of csPCa on MRI. However, even with the current version PI-RADS v2.1, there remains considerable inter/intra-reader disagreement [84,85]. Hence, many ML and DL methods have been developed for refining the differentiation between csPCa and non-csPCa, improving PI-RADS categorization, as well as possibly predicting Gleason score (GS). A recently published review [86] introduced prostate lesion classification and detection studies proposed between 2018 and February 2021. The study showed that most ML-/DL-based approaches have been conducted for the task of PCa lesion classification (either two classes or multi-classes according to lesion aggressiveness), followed by lesion detection (detection and localization of lesion). 

In this section, we summarize the studies using ML and DL for prostate lesion detection and lesion scoring with the utilization of MRI. Lesion detection was treated as differentiating csPCa with non-csPCa, as well as lesion region detection, while lesion scoring predicts PI-RADS or Gleason score /grade. The studies covered in this section are summarized in Table 3.

#### 2.4.1. Lesion Detection

The radiomics feature is defined as the quantified and high-dimensional medical imaging features extracted using ML [109]. Recent studies mainly follow the procedure that first extracts predictive radiomic features that are highly correlated with the presence of csPCa, then applies the extracted features to ML-based classifiers for distinguishing clinically significant and non-significant Pcas. For example, Algohary et al. [87] and Min et al. [88] applied the minimum redundancy maximum relevance (MRMR) method for feature extraction, while the latter further used the least absolute shrinkage and selection operator (LASSO) algorithm [110], which selects strongly correlated features via shrinking their respective regression coefficients. Wu et al. [89] applied mpMRI and radiomic features to logistic regression (LR) and SVM models for diagnosing Pca in the TZ only (AUC 0.989 and 0.949 for LR and SVM, respectively), and differentiating TZ Pca with stromal BPH (AUC 0.976). Texture analysis has the advantage of reproducibility and the ability to detect image features that may be beyond the limits of visual analysis by the human eye, and hence, can also be a useful tool to quantitatively describe the tumour heterogeneity [111]. Textured-DL [90], consisting of a 3D grey-level co-occurrence matrix extractor and a CNN to differentiate csPCa and non-csPCa, showed significantly higher AUC (0.85) than the PI-RADS-based classification (0.73). 

Very recently, Aldoj et al. [91] tested different combinations of distinct MRI (3D) sequences as input to a 3D CNN, to find the best combinations for Pca classification. The location of lesion was pre-required as an input. Eventually, the ADC, DWI, and K-trans input combination performed the best, with an AUC of 0.897. This technique has the advantage of accurately combining image inputs from different MRI pulse sequences and meta-data, something which is virtually impossible for a human reader. 

Unfortunately, DL networks on medical imaging suffer from a small number of labelled datasets, and even more so when seeking to develop algorithms to delineate the foci of clinically significant disease on MRI. Transfer learning is one means to overcome this problem, by enabling knowledge gained from large datasets in other images, to be transferred to smaller relevant datasets. 

Chen et al. [92] performed the transfer learning of two ImageNet pre-trained models for feature extraction and lesion classification. The prior feature extraction layers were frozen from further training. They obtained AUCs of 0.81 and 0.83 for the two pre-trained models. Yuan et al. [93] established three pre-trained architectures to compute features from three sequences of mpMRI. Their model achieved an accuracy of 86.92% for prostate cancer classification. Separately, Zhong et al. [94] applied a deep transfer learning (DTL)-based model using two ResNets. The AUC for the DTL model was the highest (0.726) compared with the same DL model architecture trained from scratch (0.687) and PI-RADS v2 classification (0.711). 

Other than classifying pre-defined ROIs on prostate MRI, several studies have been further developed to automatically detect and segment suspicious Pca areas. In 2015, Giannini et al. [95] used SVM to create a malignancy probability map for all voxels of the prostate, yielding a voxel-wise segmentation AUC of 0.91. Another study [96] employed a least square regression model to generate the segmentation of epithelium and lumen maps, resulting in an AUC of 0.79. To detect csPCa in low risk patients who opt for active surveillance, Arif et al. [98] used low risk Pca patients’ data to train a U-shaped 3D CNN model for lesion segmentation, achieving an AUC from 0.65 to 0.89 for lesion volumes ranging from > 0.03 to > 0.5 cc. Recently, Zhang et al. [97] combined GrowCut techniques to segment prostate cancer from MRI images, Zernik feature selection to extract features from images, and ensemble learning techniques including KNN, SVM, and MLP to determine and diagnose the lesions. They improved the accuracy by 20% compared to other methods with similar approaches (accuracy 80.97%). Seetharaman et al. [99] introduced the Stanford Prostate Cancer Network (SPCNet) to learn features specific to each sequence of MRI mapped with histopathology images, achieving a AUC of 0.86–0.89 to detect aggressive cancers and 0.75–0.85 to detect clinically significant lesions. 

#### 2.4.2. Lesion Scoring (PI-RADS and Gleason Score)

Due to the overlap between Pca lesions and false positive diseases such as BPH and prostatitis, a key tenet of PI-RADS has been to stratify lesions based on the probability of malignancy, rather than taking a binary approach to image-based diagnosis. DL approaches encode ground truth scores and MRI features for multi-class prediction. Sanford et al. [17] used Resnet34 CNN to predict the PI-RADS scores of manually segmented lesions. The predicted PI-RADS scores were associated with the expert radiologist’s, with a kappa score of 0.40. de Vente et al. [103] and Cao et al. [104] applied ordinal encoding [112], which utilizes the ordinal nature of Gleason score (GS) to CNN models for predicting the GS of Pca. A novel study in 2018 [107] determined the Gleason grade (GG) in Pca using stacked sparse autoencoders (SSAE) and a SoftMax classifier. The algorithm won first place in the PROSTATEx-2 2017 challenge, with a kappa score of 0.2326.

As mentioned earlier, lesions have different characteristics in different prostate zones. Several methods used zonal specific imaging features to different ML/DL classifiers for Pca scoring. For example, Jensen et al. [106] and de Vente et al. [105] applied a k-nearest neighbour classifier and a U-Net-based model, respectively, for GG prediction, and Wang et al. [102] employed the SVM-RFE model for PI-RADS score prediction. Their results showed that zonal specific information and radiomic features could significantly improve the prediction of aggressive scores for prostate lesions.

### 2.5. Treatment Decision Support

To patients with newly diagnosed prostate cancer, the role of MRI is to help determine the best treatment option for the patients. For example, the presence of locally advanced disease with extra-prostatic extension of disease (EPE) and the invasion of the neurovascular bundles will increase the complexity of surgical resection, but remain amendable to radiation therapy. In metastatic bone or nodal disease, systemic hormonal therapy is preferred. To date, machine learning-based methods for improving treatment prediction have been limited to determining the presence of EPE and estimating biochemical recurrence risk. The studies mentioned in this section are summarized in Table 4.

#### 2.5.1. EPE Prediction

MpMRI plays an important role in the local staging of prostate cancer. However, for the diagnosis of EPE, MRI has variable sensitivity, reportedly as low as 0.57 [130]. The accuracy of diagnosis strongly correlates with the experience level of radiologists interpreting the scans [131]. 

Recently, a radiomic-based EPE prediction has emerged. Stanzione et al. [113] predicted EPE using texture features extracted from manually segmented mpMRI maps of index lesions. Comparing different feature selection models and ML classifiers, the Bayesian network performed best with correctly classified instances of 82% and an AUC of 0.88. Similarly, Ma et al. [114] and Xu et al. [115] extracted radiomic features from mpMRI, using LASSO logistic regression for predicting extracapsular extension (ECE) and EPE, yielding AUCs of 0.83 and 0.865, respectively. Losnegård et al. [116] combined RF analysis with radiology interpretation and the MSKCC nomogram, resulting in an AUC of 0.79 for EPE prediction. Cuocolo et al. [117] applied SVM to train and test data from three different institutions, and resulted in an overall accuracy of 83%. Because the real-world implementation of ML algorithms often suffers from a drop in diagnostic performance, more extensive validation and testing of the aforementioned models are required.

#### 2.5.2. Biochemical Recurrence Prediction

Biochemical recurrence (BCR) is used for assessing the outcome of radical prostatectomy. It indicates an increase in the PSA level of patients who have undergone radiotherapy or surgery for PCa [132]. Pre-operative BCR prediction could help urologists and patients to decide on an acceptable treatment option, while post-operative BCR prediction enables an optimal surveillance. 

Currently, GS, preoperative PSA and pathological stages are the main parameters used to predict the risk of BCR. For higher accuracy and specificity, multivariate LR models such as nomograms [133] have been used to improve prediction. Although several nomograms for predicting BCR have been internationally validated, these tools have been developed with limited (often single institutional) datasets, and therefore limited accuracy [134]. MRI has been increasingly employed as an adjunct tool for improving the performance of BCR prediction. However, the clinical application of MRI can be limited by inter-reader variability, and the occasional degraded image quality from magnetic field-related artefacts. Despite these challenges, several traditional ML models have been designed to find the remarkable features in MRI for BCR prediction. 

Incorporating MRI findings into traditional regression methods has been found to be useful for BCR prediction, in some studies. Fuchsjäger et al. [118] converted MRI findings into a scoring system using Cox proportional hazards regression, and added the scores to published preoperative nomograms for BCR prediction. The model resulted in a C-index of 0.776 for 5-year BCR prediction and 0.788 for 10-year prediction. A later study [119] applied univariate and multivariate analyses using the Cox proportional hazards model to find the correlation between the PI-RADSv2 score and BCR. It is worth noting from this study that patients with PI-RADS < 4 did not suffer from BCR, indicating that PI-RADSv2 may be useful to predict PCR after RP for PCa. More recently, Capogrosso et al. [120] used 372 patients’ data for Cox regression, for assessing the association between the pre-biopsy mpMRI score and the risk of postoperative BCR. However, the authors did not demonstrate significant improvement after adding the pre-biopsy mpMRI score to the existing predictive models, probably due to the insufficient data size. A better understanding of what constitutes important MRI features would likely improve the yield that MRI brings into predictive models. 

Rather than simply relying on mpMRI scoring systems, which aggregate rather than provide sufficient emphasis on specific important imaging features, radiomic features have been employed with some promise of success. Using Cox regression analysis, Park et al. [121] analysed all clinical variables and tumour ADC data, and found that tumour ADC was the only independent predictive factor for PCR, with an AUC 0.755. The finding was later proven by Bournonne et al. [122], who extracted IBSI-compliant radiomic features [135] from mpMRIs, and found that one feature (SZEGLSZM) from ADC maps was predictive of BCR and bRFS after prostatectomy with an AUC of 0.76. Zhang et al. [123] found that the imaging-based approach using SVM was superior to LR analysis in predicting PCa outcome (accuracy 92.2% vs. 79.0%). Another study [124] employed SVM and linear discriminant analysis on radiomic features extracted from T2-weighted and ADC images, with a similar AUC of 0.73. 

There are fewer studies using DL to analyse MRI for BCR prediction. Early in 2004, Poulakis et al. [128] compared the artificial neural network (ANN) with classical regression and the Kattan nomogram model in a utilization of pelvic coil MRI, resulting in a significantly more accurate performance than the two other models (AUC 0.897 vs. 0.738, 0.728) in predicting BCR. Recently, Yan et al. [125] proposed a DL-based algorithm that consecutively extracted quantitative features from MRI and predicted BCR risk. Their model was validated in two independent cohorts, and achieved a C-index of 0.802 in both primary and validating cohorts, showing significant potential for DL-based BCR prediction. Importantly, when comparing various approaches incorporating MRI information (PI-RADS grade vs. specific imaging feature, use of DL) into these predictive models, diagnostic performance (AUC) cannot be interpreted en face, and a head-to-head comparison of the various approaches would be better to deepen our understanding of which approach to employ for clinical use.

#### 2.5.3. Histological and Outcome Predictions

Often, MRI is not able to accurately depict the extent of disease burden in PCa; notably, the presence of lymph node metastases—an important predictor of disease-free and overall survival. Kang et al. [126] compared the performance of RF and Kattan pre-operative nomogram (KN) in the prediction of organ-confined disease (OCD), EPE and lymph node metastasis using 1560 patients’ data, and found that RF may outperform KN slightly (AUC 0.75 vs. 0.69) when the positive and negative outcomes are balanced. Abdollahi et al. [127] utilized pre- and post- operative MRI radiomic features and ML classification methods to predict intensity-modulated radiation therapy (IMRT) response, GS and PCa stages, showing that non-invasive radiomic features from MR images and ML approaches are easy methods for guiding PCa diagnosis and therapy. Detailed information pertaining to tumour stage, treatment regime and subsequent treatment response can be prospectively amassed for the purposes of developing sophisticated predictive models that could allow for better individualized treatment and surveillance. This will require foresight by healthcare providers in setting up the necessary IT infrastructure to enable relevant big (structured and unstructured) data to be mined and analysed in an informative manner. 

## 3. Machine Learning Applications to Enhance Utility of Prostate MRI: Limitations

Multiple studies have shown potential in the application of ML-/DL-based methods in prostate MRI. There is good reproducibility in prostate gland and zonal segmentation, satisfactory registration between MRI and ultrasound or CT, and comparable performance with expert reads in the detection of clinically significant prostate cancer. However, applicability may be limited by relatively small validation and test datasets, as well as considerable heterogeneity across many of these studies. Furthermore, there is often difficulty obtaining ground truth validation for segmentation and image registration techniques. While the automation of these processes improves time management and allows procedurists to focus on more urgent tasks, more head-to-head comparisons, for example comparing automated vs. expert-read lesion detection and lesion scoring, are needed to compare the diagnostic performance of ML/DL-based methods with conventional interpretation, or demonstrate the added advantage of ML-/DL-based methods over manual interventions.

There remain two potential clinical questions that could be addressed by DL/ML in the future. First, patients with low-risk prostate cancer can undergo active surveillance (AS), where upfront definitive treatment such as surgery or radiotherapy is deferred until there is evidence of disease progression. Prostate MRI is increasingly being integrated into active surveillance protocols for this group of patients, where the aim is to determine radiologic change between interval surveillance MRI scans which would suggest disease progression [136]. ML has been utilised to predict the probability of disease progression for patients on AS, based on clinical criteria such as age, family history, serum PSA, and tumour volume [137]. However, to our knowledge, ML/DL methods in prostate MRI have yet to be incorporated into active surveillance strategies. The detection of a significant change between surveillance MRI can be challenging for even the most experienced radiologist, and ML/DL methods would be very helpful in this setting. Second, patients diagnosed with BCR often undergo a prostate MRI to evaluate for local recurrence. Post-treatment MRI appearance in this group of patients who have either undergone surgery, radiotherapy or focal therapy will differ considerably from pre-treatment MRI. To our knowledge, there are no published studies evaluating ML/DL in a post-treatment MRI scan for the detection of local recurrence. The assessment for local recurrence can be challenging for radiologists, given the variable morphological features on MRI. ML/DL applications will also be very helpful in this setting.

## 4. Future Opportunities

With the above advances in ML techniques, it is foreseeable that a combination of various approaches could ultimately reproduce a radiologist’s role in MRI prostate interpretation. Alkadi et al. [101] developed a DL encoder–decoder architecture that could concurrently segment the prostate, define its anatomical structure, and mark out suspicious lesions. With an employment of 3D spatial information from MR series, their accuracy for cancer detection was 0.894. Recently, Mehralivand et al. [100] proposed a cascaded DL model for lesion detection and scoring on bpMRI. The model contained a 3-D UNet-based network that automatically detected and segmented prostate MRI lesions, and two 3D residual networks that made 4-class classification to predict PI-RADS categories. The mean DSC for lesion segmentation was 0.359. Hoar et al. [54] compared several ML and comprehensive DL models, and found that the combination of transfer learning and test time augmentation resulted in a significant improvement (DSC 0.59, AUC 0.93) in CNN lesion segmentation for a small set of mpMRI data comprising 154 patients. Whereas image segmentation and registration are relatively simpler tasks, much validation work remains, in order to develop these lesion detection and characterization prototypes into diagnostic tools that can meet regulatory standards and gain adoption into mainstream clinical practice.

### Federated Learning

The data sharing, privacy and security policy may limit the ML model training that requires large (centralized) datasets. In the past few years, federated learning (FL) attracted much attention and many interests in the area where data sharing is a concern. FL is the technology enabling collaborative learning with de-centralized data [138]. However, it is still in the early stage, and challenges remain for medical applications [139], mainly due to policy and constraints on the access to learning infrastructure. The efficacy of the FL model has to be validated with more real-world studies. A recent survey on FL [140] summarises the related areas on data distribution, privacy protection, machine learning model aggregation, and communication. In applications of prostate imaging, recent works focus only on prostate segmentation [42,141], showing outperforming results over models using single data sources. A study to use FL for COVID-19 outcome predictions based on EMR data across over 20 countries [142] shows the potential to collectively use multiple data sources for medical applications. Commercially, there are platforms that researchers can use, as well as open platforms available; examples include Nvidia Clara [143], Tensorflow Federated [144], IBM FL [145], OpenFL [146], FATE [147], XayNey [148], Baidu FL [149] et al. We anticipate that federated learning could accelerate the development of DL algorithms in domains where large datasets are not feasible from single institutions alone, such as in the case of MRI prostate.

## 5. Conclusions

Rapid advancements in ML and DL have led to a recent surge in interest in the domain of computer vision in medical imaging. Prostate MRI is an ideal imaging modality for DL applications, given that it is the mainstay of lesion detection, and in line with a trend towards targeted biopsy and local ablative therapies to improve clinical outcomes. It has remarkable potential to increase the manpower productivity of radiologists and radiation oncologists over the manual segmentation of the organ, reduce interobserver variability in lesion detection and cross-modality image co-registration, and advance the accuracy of PIRADS through predictive analytics and federated learning for risk stratification, and the individualized care of patients at risk and with prostate cancer. 

## Figures and Tables

**Table 2:** Machine learning-based MR image registration methods. The abbreviations are shown below ^2^.

Publication Year	Approach	Registration Type	Registration Modalities	ML/DL Method	Auto-Seg	Sample Size	CV	Results					Refs.
								TRE (mm)	DSC%	MSD (mm)	HD (mm)	Error (%)	
2002	Knowledge-based	Deformable	MRI–TRUS	homogeneous Mooney-Rivlin model, Linear least squares fitting	N	25 simulations of TRUS	No	-	-	-	-	26.7	[66]
2011	Knowledge-based	non-rigid, deformable	MRI–TRUS	PCA	N	5 patients	Leave-one-out	5.8	-	-	-	-	[67]
2012	Knowledge-based	non-rigid, deformable	MRI–TRUS	PCA	N	8 patients	Leave-one-out	2.4	-	-	-	-	[68]
2016	Knowledge-based	non-rigid, deformable	MRI–TRUS	PCA, surface point matching	N	1 MRI dataset and 60 TRUS datasets	Leave-one-out	1.44	-	-	-	-	[69]
2018	Weakly supervised	Deformable	MRI–TRUS	CNN	N	111 pairs	10-fold	9.4	73	-	-	-	[70]
2018	Weakly supervised	non-rigid, deformable	MRI–TRUS	CNN	N	76 patients	12-fold	3.6	87	-	-	-	[71]
2018	Unsupervised	Affine	MRI–TRUS	GAN, CNN	N	763 pairs	No	3.48	-	-	-	-	[72]
2020	Weakly supervised	Affine and nonrigid, deformable	MRI–TRUS	FCN, 3D UNet	Y	36 pairs	Leave-one-out	2.53	91	0.88	4.41	-	[73]
2021	Weakly supervised	Deformable	MRI–TRUS	3D UNet	Y	288 patients	No	-	87	-	7.21		[74]
2020	Supervised	Rigid, Deformable	MRI–TRUS	UNet, CNN	Y	12 patients	No	2.99	-	-	-	-	[75]
2018	Knowledge-based and DL	Non-rigid, deformable	MRI–TRUS	3D encoder-decoder	N	108 pairs	12-fold	6.3	82	-	-	-	[76]
2020	Knowledge-based and DL	non-rigid, deformable	MRI–TRUS	CNN, 3D Point Cloud	Y	50 patients	Leave-one-out	1.57	94	0.90	2.96	-	[77]
2019	Supervised	Rigid, deformable	MRI–CT	RF based on an Auto-context model	N	17 treatment plans from 10 patients	No	-	-	-	-	<1	[78]
2020	Knowledge-based	Rigid, Affine, and Deformable	MRI–histology images	-	N	157 patients	No	-	97	-	1.99	-	[79]
2021	Knowledge-based	Rigid, deformable	MRI–CBCT	CNN, 3D Point Cloud	Y	50 patients	5-fold	2.68	93	1.66	-	-	[80]
2021	Unsupervised	Affine, Deformable	MRI–histology image	CNN	N	99 patients (training), 53 patients (test)	No	-	97.5, 96.1, 96.7	-	1.72, 1.98, 1.96	-	[81]
2017	Unsupervised	Rigid, affine, deformable	MRI–histology image	Multi-image super-resolution GAN	N	533 patients	5-fold	-	95 (prostate), 68 (cancer)	-	-	-	[59]

^2^ Auto-Seg = auto-segmentation, CV = cross-validation, TRE = target registration error, DSC = dice similarity coefficient, MSD = mean surface distance, HD = Hausdorff distance, Refs. = reference, MRI–TRUS = MRI–transrectal ultrasound, - = not reported.

**Table 3:** Machine learning methods for lesion detection and characterization. The abbreviations are shown below ^3^.

Publication Year	Application	Method	Serum PSA (ng/mL)	Prostate Zone	Data Source	MRI Sequence(s)	Sample Sizes:			CV	Ground-Truth	Non-MRI Data Features (If Any)	Results			Refs.
							Train	Val	Test				Acc, AUC (%)	Ssv, Spc (%)	Kap, DSC (%)	
2018	Detecting csPCa in AS patients	MRMR, QDA, RF, SVM	6.96 ± 5.8	WG	Pv	T2w, ADC	31	-	25	3-fold (training)	PI-RADS score and biopsy	-	72, -	-	-	[87]
2019	Differentiating csPCa and non-cs PCa	MRMR and LASSO algorithm	>10	WG	Pv	T1w, T2w, DWI, ADC	187	-	93	10-fold	Gleason Score	-	-, 82.3	84.1, 72.7	-	[88]
2019	Differentiating TZ Pca from BPH	Logistic Regression and SVM	-	TZ	Pv	T2w, ADC	105	-		No	-	-	-, 98.9	93.2, 98.4	84 (tumour), 87 (BPH)	[89]
2021	Prediction of csPCa (PI-RADS ≥ 4)	Textured-DL and CNN	4.7–8.7	WG	Pv	T2w, ADC	239	42	121	No	PI-RADS score	-	-, 85	-, 70	-	[90]
2020	Differentiating csPCA and non-cs Pca	3D CNN	-	WG	PROx	ADC, DWI, K-trans (from DCE)	175	-	25	8-fold	PI-RADS score	Location of lesion center	-, 89.7	81.9, 86.1	-	[91]
2017	Differentiating csPCa and non-cs Pca	Transfer learning, ImageNet	-	WG	PROx	T2W, DWI, ADC, DCE	330	-	208	k-fold	PI-RADS score	-	-, 83	-	-	[92]
2019	Classifying low-grade and high-grade Pca	Transfer learning, AlexNet NN	-	WG	Pv, PROx-2	T2w, ADC	110	66	44	No	Gleason Score	-	86.92, -	-	-	[93]
2019	Prediction of csPCa (PI-RADS ≥ 4)	Transfer learning	7.9 ± 2.5	WG	Pv	T2w, ADC	169		47	No	PI-RADS score	Zonal information	72.3, 72.6	63.6, 80	-	[94]
2015	PZ cancer detection	Regression, SVM	4.9–8.6	PZ	Pv	T2w, ADC	56		56	Yes	prostectomy	-	-, 91	97	-	[95]
2018	Predictive maps of epithelium and lumen density	Least square regression	-	WG	Pv	T2w, ADC, ECE	20	-	19	No	prostectomy	-	-, 77(epithelium); 84 (lumen)		-	[96]
2021	Pca detection and segmentation	Growcut, Zernik, KNN, SVM, MLP	-	PZ, TZ	Pv	T2w	217	-	54	No	prostectomy	Clinical and histopathological variables	80.97, -	-	79	[97]
2020	Pca detection and segmentation	3D CNN	-	WG	Pv	T2w, DWI, ADC	116	-	155	3-fold	biopsy	Location of lesion	-, 0.65–0.89	82–92, 43–76	-	[98]
2021	Pca differentiation and segmentation	SPCNet	6.8–7.1	WG	Pv	T2w, ADC	102	-	332	5-fold	prostectomy	-	-, 0.75–0.85	-	-	[99]
2021	Pca detection and classification	Cascaded DL	4.7–9.9	WG	Pv, PROx	T2w, ADC	1290	-	150	5-fold	PI-RADS score	-	30.8, -	56.1, -	35.9	[100]
2021	Pca segmentation	Transfer learning, CNN, Test time augmentation	2.1–18	WG	Pv, PROx	T2w, DWI and DCE	16, 16	-	16	Leave-one-out	prostectomy	-	-	-	59	[54]
2018	Pca segmentation	Encoder–decoder CNN	-	WG, PZ, CG	I2CVB	T2w	1413	236	707	10-fold	Radiologist segmented results	-	89.4, -	-	-	[101]
2017	Improve PI-RADS v2	RBF-SVM, SVM-RFE	12.5–56.1	WG, TZ, PZ	Pv	T2w, DWI, DCE	97	-	-	Leave-one-out	PI-RAD scores	-	-, 98.3 (PZ); 96.8 (TZ)	94.4 (PZ); 91.6 (TZ), 97.7 (PZ); 95.5 (TZ)	-	[102]
2020	Prediction of PI-RADS v2 Score	Resnet34 CNN	-	WG	Pv, PROX	T2W, DWI, ADC, DCE	482	137	68	No	PI-RADS score	-	-	-	40, -	[17]
2019	Pca detection, prediction of GGG score	Unet, batch normalization, ordinal regression	-	WG	PROX-2	T2w, ADC	99	-	63	5-fold	Gleason score	-	-	-	32.1	[103]
2019	Pca segmentation, prediction of GS Score	multi-class CNN (Deeplab)	-	WG	Pv	T2w, DWI	417	-	-	5-fold	Gleason score	-	-, 80.9	88.8, -	-	[104]
2021	Prediction of GGG score	Unet, ordinal regression	-	WG, TZ, PZ	PROX-2	T2W, DWI, ADC	112	-	70	5-fold	GGG	Zonal information	-	-	13, 37	[105]
2019	Prediction of GGG score	KNN	-	TZ, PZ	Pv	T2w, DCE, DWI, ADC	112	-	70	3-fold	GGG	Texture features, zonal information	-, 92 (PZ); 87 (TZ)	-	-	[106]
2018	Prediction of GGG score	Stacked sparse autoencoders	-	WG	PROX-2	T2w, DWI, ADC,	112	-	70	3-fold	GGG	Hand-crafted texture features	47.3, -	-	27.72, -	[107]
2021	Lesion detection and classification	Cascaded DL	4.7–9.9	WG	Pv, PROX	T2w, ADC	1290	-	150	5-fold	PI-RADS score	-	30.8, -	56.1, -	-, 35.9	[100]

^3^ Val = validation, CV = cross-validation, Acc = accuracy, AUC = area under ROC curve, Ssv = sensitivity, Spc = specificity, Kap = Kappa score, DSC = dice similarity coefficient, Refs. = reference, - = not reported. csPCa = clinically-significant prostate cancer, GGG = Gleason grade group. For prostate zones, WG = whole gland, PZ = peripheral zone, TZ = transition zone. For data source, Pv = private, PROx = PROSATETx Challenge [15], PROx-2: PROSATETx-2 challenge [15], I2CVB: I2CVB Benchmark dataset [108].

**Table 4:** Machine learning methods for treatment aiding. The abbreviations are shown below ^4^.

Publication Year	Application	Method	Input Feature	Sample Size	Ground-Truth	MRI Sequence (s)	CV	Results			Refs.
								Acc (%)	AUC (%)	C-Index	
2019	EPE Detection	Bayesian Network, Texture analysis	Index lesions from biparametric MRI	39	Prostectomy	T2w, ADC	No	82	88	-	[113]
2020	ECE Prediction	LASSO regression	ROIs of T2W images	119	Prostectomy	T2w, DWI, DCE	10-fold	-	82.1	-	[114]
2020	EPE Detection	LASSO regression	Radiomic features, patients’ clinical and pathological variables	115	Prostectomy	T2w, ADC, DWI, DCE	No	81.8	86.5	-	[115]
2020	EPE Prediction	Combination of RF model, radiology interpretation and clinical nomogram	MR radiomic features	228	Prostectomy	T1w, T2w, DWI, DCE	10-fold	-	79	-	[116]
2021	EPE Detection	SVM	Radiomic feature from MRI index lesions	193	Prostectomy	T2w, ADC	10-fold	79	-	-	[117]
2009	BCR Prediction	Cox regression	GS and clinical variales	610	BCR defined by NCCN guideline	T2w, DWI, ADC, DCE	No	-	-	0.776 (5-year), 0.788 (10-year)	[118]
2015	BCR Prediction	Univariate and multivariate analyses using Cox’s proportional hazards model	PI-RADSv2 score, surgical parameters	158	Two consecutive PSA ≥ 0.2 ng/mL	T2w, DWI, DCE	No	-	-	-	[119]
2019	pre-biopsy mpMRI to improve preoperative risk model	Cox regression	pre-biopsy mpMRI score	372	Two consecutive PSA ≥ 0.1 ng/mL	T1w, T2w	No	-	-	-	[120]
2010	BCR Prediction	Univariate and multivariate analyses	Clinical variables and tumour ADC data	158	PSA ≥ 0.2 ng/mL	ADC, DWI	No	-	75.5	-	[121]
2019	BCR and bRFS Prediction	Univariate and multivariate Cox regression	IBSI-compliant radiomic features	107	Two consecutive PSA ≥ 0.2 ng/mL	T2w, ADC	No	-	76	-	[122]
2016	BCR Prediction	SVM	Clinicopathologic and bpMRI variables	205	PSA ≥ 0.2 ng/mL	T2w, DWI, DCE	5-fold	92.2	-	-	[123]
2018	Identify predictive radiomic features for BCR	SVM, Linear discriminant analysis and RF	Radiomic features from pretreatment bpMRI	120	PSA > 0.2 ng/mL (post-RP) and PSA >2 ng/mL (post-RT)	T2w, ADC	3-fold	-	73	-	[124]
2021	BCR Prediction	Radiomic-based DL	Quantitative features of MRI	485	PSA ≥ 0.2 ng/mL	T1w, T2w, DWI, ADC	No	-	-	0.802	[125]
2018	Post-Prostatectomy Pathology prediction	RF	Demographics, PSA trends, and location-specific biopsy findings	1560	Prostatectomy	-	-	-	75 (OCD), 73 (ECE), 64 (pN+)	-	[126]
2019	IMRT response prediction	Univariate radiomic analysis, ML classification models	pre-/post-IMRT mpMRI radiomic features	33	Change of ADC values before and after IMRT.	T2w, ADC	10-fold	-	63.2	-	[127]
2004	BCR Prediction	ANN	MRI findings, PSA, biopsy Gleason score	210	PSA level ≥ 0.1 ng/mL	T2w, DWI, ADC, DCE	5-fold	-	89.7	-	[128]

^4^ CV = cross-validation, Acc = accuracy, AUC = area under ROC curve, C-Index = concordance index [129], Refs. = reference, - = not reported. bRFS = biochemical recurrence free survival, RP = radical prostatectomy.

## Data Availability

Open search internet, A*Star digital library.
